# Case Report: First case of HIV, hepatitis B, hepatitis C, and Vibrio vulnificus coinfection

**DOI:** 10.3389/fcimb.2023.1290636

**Published:** 2023-12-11

**Authors:** Huimin Zeng, Jialong Guan, Chudan Liang, Yulong Wang, Lizhi Feng, Han Zhao, Linjin Fan, Xiaofeng Yang, Nenglang Pan, Zequn Wang, Haolan He, Zhimin Chen, Jun Qian, Yueping Li, Linna Liu

**Affiliations:** ^1^ Institute of Infectious Diseases, Guangzhou Eighth People’s Hospital, Guangzhou Medical University, Guangzhou, China; ^2^ Zhongshan School of Medicine, Sun Yat-sen University, Guangzhou, China; ^3^ Key Laboratory of Tropical Disease Control (Sun Yat-sen University), Ministry of Education, Guangzhou, China

**Keywords:** HIV, hepatitis B, hepatitis C, Vibrio vulnificus, hamster models

## Abstract

Our patient, a 48-year-old man from Guangdong’s coastal region, worked selling and processing oysters and other seafood. He started experiencing swelling and pain in his left knee on October 4, 2022, and they got worse over time. The findings of mNGS test showed Vibrio vulnificus infection. The patient had AIDS, hepatitis A and hepatitis B concurrently. He was admitted to the hospital’s intensive care unit (ICU) for treatment as his symptoms worsened. We refrained from performing an amputation because the family members expressed a desire to keep the limb. The limb was managed with regular dressing changes, thorough debridement, wound closure, ongoing VSD drainage, and local antibiotic irrigation. The patient’s organ function eventually returned to normal, and the systemic infection got better. On November 1, the wound’s new granulation tissue had grown well and had gradually crept to cover 80% of the wound. The tissue’s blood flow had also improved, indicating a trend of growth and healing.

## 1 Introduction

In 2021, there were 37.7 million people living with HIV, 296 million people living with chronic hepatitis B virus infection, and 58 million people living with chronic hepatitis C virus infection (WHO, 2021). Among HIV-positive individuals, 7.4% (2.7 million) also had a coinfection with HBV, and 6.2% (2.3 million) had a coinfection with HCV (Arora et al., 2021). Hepatitis viruses were found in 94.4% of HIV patients. Anti-HCV was positive in 72% of cases, triple infections were seen in 7.9% of cases, and viral indicators for HBsAg were present in 14.5% of cases (Mohammadi et al., 2009; Masroor et al., 2021). However, it is challenging to create a therapeutic strategy when these viruses co-occur with Vibrio vulnificus infection. We describe a case of coinfection with all four pathogens caused by a history of intravenous drug use and talk about the therapeutic options used in this instance.

V. vulnificus is an opportunistic pathogen with high mortality that causes wound infections that can rapidly lead to septicemia (Baker-Austin et al., 2018). Case fatality rates are higher than 50% for primary septicemia, and death typically occurs within 72 hours of hospitalization (Yun and Kim, 2018). Both ingestion of contaminated fish and shellfish as well as skin contact with contaminated seawater are ways that V. vulnificus might spread (Daniels, 2011). A traumatic infection may be fatal in susceptible individuals, leading to a severe wound infection and even septic shock that may necessitate amputation. The most common clinical subtypes of V. vulnificus infection include primary sepsis, traumatic infection, and gastroenteritis. These subtypes are characterized by rapid symptom progression and signs of multiple organ dysfunction syndrome (MODS) symptoms (Leng et al., 2019).

There haven’t been any published reports of HIV, hepatitis B, hepatitis C, and Vibrio vulnificus coinfection as of yet. As a result, we describe the clinical characteristics and diagnostic process of the first instance of reported coinfection with the aforementioned four viruses in this study.

## 2 Our study client

Our patient, a 48-year-old man from Guangdong’s coastal region, worked selling and processing oysters and other seafood. He started experiencing swelling and pain in his left knee on October 4, 2022, and they got worse over time. The skin temperature rose, there was visible discomfort, and the surrounding area was red and swollen. The following day, his right leg began to hurt and feel numb. He also progressively began to develop a huge fusion erythema with a low skin temperature. Then, a number of distinct blisters developed on his legs. His lower extremities started to hurt more on October 5,particularly his left knee. The patient had a 10-year history of intravenous drug addiction and no prior history of leg damage prior to the commencement of the condition.

## 3 Case representation and discussion

The patient was infected with HIV/HBV/HCV. He claimed that despite receiving an HIV infection in 2002, he did not start antiviral therapy. In 2016, he started antiviral treatment (3TC+TDF+EFV), which was changed to 3TC+TDF+NVP due to hallucinations and abnormal mental behavior. After using the medication for about a year, he stopped. In 2018, his HIV RNA level reached 1×10^5^ IU/ml, and his CD4^+^ concentration reached 400 cells/µl in September 2019. The original protocol was changed to 3TC+TDF+LPV/r after 15 days of therapy, and on December 18, 2019 it was changed to 3TC+TDF+RAL because of gastrointestinal symptoms. The patient was utilizing FTC/TAF+RAL on January 23, 2020, and he had low viremia, inadequate virus response, and the potential for drug resistance was taken into consideration (no drug resistance test was done).

**Figure 1:**
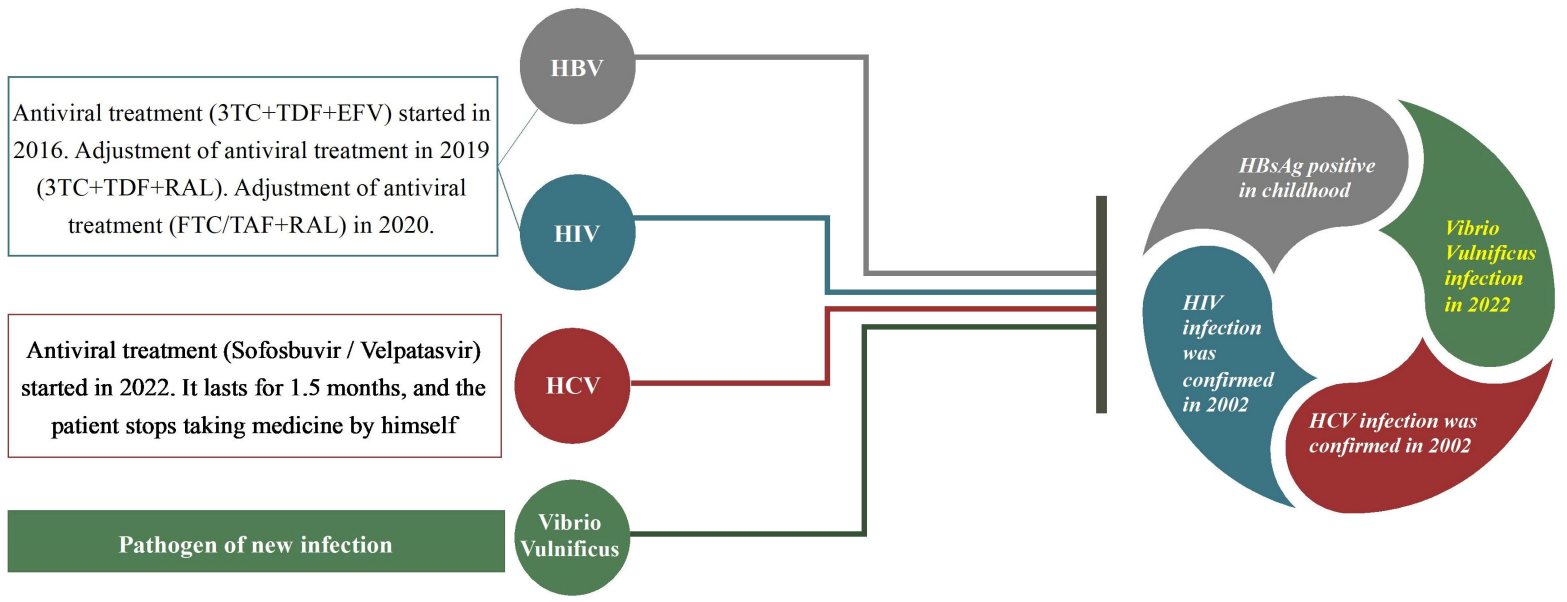
A patient had coinfection with HIV, hepatitis B (HBV), hepatitis C (HCV) and Vibrio vulnificus and he had received some treatment previously.

The patient stated that he developed HBsAg positivity when he was young, and on October 21, 2019, his HBV DNA level reached 1.14 × 10^3^ IU/ml. He had a history of chronic hepatitis C, and in 2002, he was found to be infected with HCV. His HCV DNA level reached 1 × 10^6^ IU/ml in August 2019. In August 2022, he began anti-HCV treatment (sophobuvir/vipatavir). The treatment lasted approximately 1.5 months, and then he stopped taking the drugs. The patient’s viral infection and treatment regimen before admission are presented in **Figure 1**.

On October 7, the patient was transferred to Guangzhou Eighth People’s Hospital of Guangzhou Medical University for treatment. When he arrived at the hospital at 8:35, his left knee and right calf were swollen and painful, he was tired, restless, had little urine output, low temperature (36 °C), low blood pressure (71/52 mmHg), and was short of breath (35 times/minute). During the physical examination, he had a big erythema on his right leg, which was similar to a scalded skin lesion, approximately 22 cm × 8 cm. There were multiple local blisters with defined boundaries, low skin temperature, and mild pulse of the dorsalis pedis artery. The skin temperature was rising, and there was obvious tenderness in his left knee joint, which was red and swollen.

The laboratory examination showed severe acidosis (pH 7.169), hyper lactic acidemia (Lac 21 mmol/L), acute renal insufficiency, liver insufficiency, elevated muscle enzymes, elevated hemogram (WBC 29.31 × 10e9/L, CRP 122.96 mg/L), thrombocytopenia (PLT 39~64 × 10e9/L), abnormal coagulation function, etc. Blood samples and blister fluid were taken two hours after arrival in order to perform metagenomic next-generation sequencing (mNGS). Considering the rapid progression of the patient’s condition and multiple organ dysfunction (liver, kidney, heart, lung), he was transferred to the ICU for monitoring and treatment.

The patient received liquid resuscitation and organ function support, and treated for septic shock and MODS after being moved to the ICU. Meropenem was administered intravenously after doxycycline. The patient was later switched to anti-infective medications like cefotaxime sulbactam sodium due to his enduring high fever and declining liver function. In addition to administering analgesics, sedative therapy, nutritional support, and other treatments, we used CRRT to reduce inflammatory variables and an oXiris filter to absorb endotoxins and cytokines (the filter was changed every 12 hours).

His lactic acid level dropped to 2.0 at 14 hours after admission, but the patient’s right lower limb darkened and progressed to dark brown, with more vesicles dispersed on the surface, and they partially fused into large, tense, purplish-black blood blisters. Simultaneously, erythema appeared on the inflamed area of the left knee joint. The epidermis on the right lower leg simply peeled off on October 8 after the blisters merged and produced a significant amount of exudate. The epidermis was dark gray, and the tissue under the epidermis was gray white. The soft tissue changes of the limbs were gangrenous.

On October 9, the mNGS results showed a Vibrio vulnificus infection. In particular, a library of DNA samples was created using the Universal Plus DNA Library Prep Kit for MGI (VAHTS, NDM617), and MGISEQ-200RS was used for detection. After sequencing and quality control and screening, it was compared with the independently constructed microbial genome database Richardson Diagnostics DB v1.0, and then the classification database in NCBI taxonomy was used for species identification.

**Figure 2:**
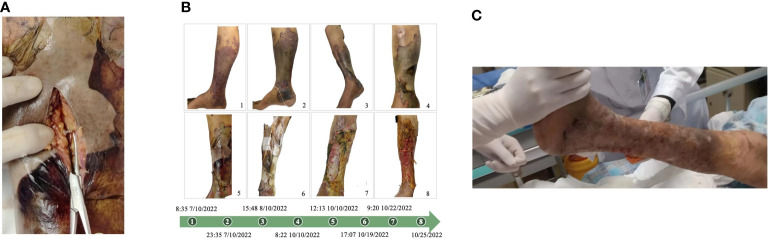
**(A)** Perform incision decompression to treat the infected part. At the infection site, deep tissue vascular embolism, necrotizing fasciitis, and a large amount of exudation can be seen. **(B)** Clinical manifestations of the patient’s right lower limb from October 7 to October 25, 2022. **(C)** The patient healed well after skin grafting, with stable clinical signs, and left hospital on November 26, 2022.

According to the diagnosis and treatment guidelines, we conducted anti-infection treatment (meropenem+levofloxacin). He received open decompression therapy on October 10 because the skin on his right lower limb was dark gray and exudative. Deep vascular embolism, necrotizing fasciitis, and a significant amount of exudate in the skin tissue were all visible during the procedure (**Figure 2A**). Debridement and hydrogen peroxide washing of the wound, VSD negative pressure device continuous drainage, and physiological saline irrigation were performed. Considering the possibility of skin and soft tissue infection caused by Gram-positive cocci, teicoplanin was added. On October 12, the drug sensitivity report indicated that it was a sensitive strain, and the wound secretion gradually changed from clear exudate/mild blood liquid to a purulent secretion, with a strong odor, increased infection, and severe necrosis of the muscle and other soft tissues.

Amputation treatment was assessed in consultation with the orthopedics department’s clinical experts, and the anesthesia department was asked to assess the risk of anesthesia. The patient’s vital signs were unstable, his respiratory oxygenation was less than 200, the risk of the operation and anesthesia was high, and his circulation was unstable. The family members expressed their desire to save the limb, so we did not perform amputation. The limb was treated with timely dressing changes, repeated debridement, wound healing, continuous VSD drainage, and local irrigation with sensitive antibiotics. The patient’s organ function gradually recovered, and the systemic infection improved. On November 1, the wound’s new granulation tissue had grown well and progressively crept to cover 80% of the wound. The tissue’s blood flow had also improved, indicating a trend of growth and healing. **Figure 2B** depicts the changes in the patient’s right lower limb injuries in chronological order. The patient healed well following skin grafting, with stable clinical indications, and left hospital on November 26 (**Figure 2C**).

The patient was discharged 42 days after the surgery. 291 days later, we followed up the patient and found that he had returned to normal work and life, and had good self-care ability (ADL score of 14 on the Daily Living Ability Scale). After the wound heals, there is no discernible difference in the injured limb’s functionality between the patient and before the disease started once the wound had healed. Skin scarring are the main aftereffects.

Overall, the following elements may be primarily responsible for this patient’s successful treatment:

(1) Early comprehensive therapy of sepsis involves CRRT+Oxiris filters to remove inflammatory factors, in order to prevent the broad damage produced by cytokine cascade events within the first 48 hours.
(2) Active surgical intervention. Early diagnosis and emergency surgical debridement of necrotic soft tissue infection (NSTI) remain the cornerstone of treatment. The earlier the debridement surgery is performed, the better the treatment effect, and the more it can promote a benign outcome of the condition. For this patient with necrotizing fasciitis caused by Vibrio vulnificus, it is necessary to build a multidisciplinary diagnosis and treatment team and invite a surgeon competent in infectious wound management for the patient.
(3) Postoperative wound management and VSD drainage in emergency surgery to promote tissue regeneration. Vacuum Sealing Drain (VSD) is a technique pioneered by German trauma surgeon Dr. Fleischmann in 1992 and widely utilized in the field of trauma surgery. Negative pressure can cause exudation from the wound to the dressing, reducing the opportunity for bacteria to invade the tissue, reducing the accumulation of inflammatory mediators and lactic acid, reducing tissue edema, improving wound microcirculation, and promoting granulation tissue proliferation.

Vibrio vulnificus infection usually progresses rapidly. The local lesions advance swiftly, skin and muscle damage can occur within 24-48 hours, and the lesions can be worsened and expand within a few hours. At the same time, the patient’s overall state deteriorates rapidly, most of them develop shock within 24-48 hours, and MODS rapidly appears. Therefore, Early detection is therefore essential for tailored follow-up care.

Most cased of Vibrio vulnificus infection begin with primary sepsis caused by wound infection and gastrointestinal tract infections. The disease has a fairly well-defined high-risk population as well as an area and season of onset. The receiving doctor can quickly match the patients with available medical resources after screening patients with Rich scores, precheck and triage. Jie-Heng Liang (2021) reported the case of a 53-year-old man who was infected with V. vulnificus as a result of a bee sting. The patient had no history of contact with the sea, fresh water, aquatic organisms or products. This case study suggests that V. vulnificus may be spread by insects (Liang et al., 2021). A patient with fatal V. vulnificus encephalitis and localized brain lesions was described by Yong Woo Lee in 2021. In actuality, meningoencephalitis is infrequently caused by this condition (Lee and Kim, 2021).

## 4 Conclusion

In conclusion, we should attempt to provide empirical anti-infection treatment and early medication intervention within 1-2 hours and make an early clinical diagnosis of these individuals based on their epidemiological characteristics and clinical symptoms. When diagnosis and treatment are started 72 hours after symptoms appear, the case fatality rate might reach 100% (Klontz et al., 1988). Therefore, to prevent misdiagnosis, we should be aware of other infection-causing agents and clinical symptoms.

## Data availability statement

The original contributions presented in the study are included in the article/supplementary material. Further inquiries can be directed to the corresponding authors.

## Ethics statement

The studies involving humans were approved by ethics committee of the Guangzhou Medical University Afflicted Eighth Hospital. The studies were conducted in accordance with the local legislation and institutional requirements. The participants provided their written informed consent to participate in this study. Written informed consent was obtained from the individual(s) for the publication of any potentially identifiable images or data included in this article.

## Author contributions

HMZ: Writing – original draft. JG: Writing – original draft, Data curation. CL: Data curation, Writing – original draft. YW: Data curation, Writing – original draft. LZF: Data curation, Writing – original draft. HZ: Data curation, Writing – original draft. LJF: Data curation, Writing – original draft. XY: Data curation, Writing – original draft. NP: Formal Analysis, Writing – original draft. ZW: Formal Analysis, Writing – original draft. HH: Data curation, Writing – original draft. ZC: Data curation, Writing – original draft. JQ: Data curation, Funding acquisition, Writing – review & editing. YL: Conceptualization, Funding acquisition, Writing – review & editing. LL: Conceptualization, Funding acquisition, Methodology, Supervision, Validation, Visualization, Writing – review & editing.

## References

[B1] Arora U. Garg P. Agarwal S. Nischal N. Shalimar Wig N. (2021). Complexities in the treatment of coinfection with HIV, hepatitis B, hepatitis C, and tuberculosis. Lancet Infect. Dis. 21 (12), e399–e406. doi: 10.1016/s1473-3099(20)30765-9 34023004

[B2] Baker-Austin C. Oliver J. D. Alam M. Ali A. Waldor M. K. Qadri F. . (2018). Vibrio spp. infections. Nat. Rev. Dis. Primers 4 (1), 8. doi: 10.1038/s41572-018-0005-8 30002421

[B3] Daniels N. A. (2011). Vibrio vulnificus oysters: pearls and perils. Clin. Infect. Dis. 52 (6), 788–792. doi: 10.1093/cid/ciq251 21367733

[B4] Klontz K. C. Lieb S. Schreiber M. Janowski H. T. Baldy L. M. Gunn R. A. (1988). Syndromes of Vibrio vulnificus infections. Clinical and epidemiologic features in Florida cases 1981-1987. Ann. Intern. Med. 109 (4), 318–323. doi: 10.7326/0003-4819-109-4-318 3260760

[B5] Lee Y. W. Kim J. H. (2021). A case of vibrio vulnificus infection presenting with fatal bacterial encephalitis. J. Clin. Neurol. 17 (1), 128–130. doi: 10.3988/jcn.2021.17.1.128 33480208 PMC7840328

[B6] Leng F. Lin S. Wu W. Zhang J. Song J. Zhong M. (2019). Epidemiology, pathogenetic mechanism, clinical characteristics, and treatment of Vibrio vulnificus infection: a case report and literature review. Eur. J. Clin. Microbiol. Infect. Dis. 38 (11), 1999–2004. doi: 10.1007/s10096-019-03629-5 31325061

[B7] Liang J. H. Liang W. H. Deng Y. Q. Fu Z. G. Deng J. L. Chen Y. H. (2021). Vibrio vulnificus infection attributed to bee sting: a case report. Emerg. Microbes Infect. 10 (1), 1890–1895. doi: 10.1080/22221751.2021.1977589 34487488 PMC8477795

[B8] Masroor H. Qazi U. M. Masroor A. Saleem A. Khalil G. Abbas K. (2021). Coinfection of hepatitis B and hepatitis C virus in patients with human immunodeficiency virus. Cureus 13 (7), e16474. doi: 10.7759/cureus.16474 34466302 PMC8396414

[B9] Mohammadi M. Talei G. Sheikhian A. Ebrahimzade F. Pournia Y. Ghasemi E. . (2009). Survey of both hepatitis B virus (HBsAg) and hepatitis C virus (HCV-Ab) coinfection among HIV positive patients. Virol. J. 6, 202. doi: 10.1186/1743-422x-6-202 19922624 PMC2785785

[B10] WHO (2021). Global progress report on HIV, viral hepatitis and sexually transmitted infections. Available at: https://www.who.int/publications/i/item/9789240027077.

[B11] Yun N. R. Kim D. M. (2018). Vibrio vulnificus infection: a persistent threat to public health. Korean J. Intern. Med. 33 (6), 1070–1078. doi: 10.3904/kjim.2018.159 29898575 PMC6234401

